# Chinese translation norms for 1,429 English words

**DOI:** 10.3758/s13428-016-0761-x

**Published:** 2016-06-20

**Authors:** Yun Wen, Walter J. B. van Heuven

**Affiliations:** School of Psychology, University of Nottingham, University Park, Nottingham, NG7 2RD UK

**Keywords:** English to Chinese translation norms, Translation ambiguity, Chinese-English bilinguals

## Abstract

We present Chinese translation norms for 1,429 English words. Chinese-English bilinguals (N = 28) were asked to provide the first Chinese translation that came to mind for 1,429 English words. The results revealed that 71 % of the English words received more than one correct translation indicating the large amount of translation ambiguity when translating from English to Chinese. The relationship between translation ambiguity and word frequency, concreteness and language proficiency was investigated. Although the significant correlations were not strong, results revealed that English word frequency was positively correlated with the number of alternative translations, whereas English word concreteness was negatively correlated with the number of translations. Importantly, regression analyses showed that the number of Chinese translations was predicted by word frequency and concreteness. Furthermore, an interaction between these predictors revealed that the number of translations was more affected by word frequency for more concrete words than for less concrete words. In addition, mixed-effects modelling showed that word frequency, concreteness and English language proficiency were all significant predictors of whether or not a dominant translation was provided. Finally, correlations between the word frequencies of English words and their Chinese dominant translations were higher for translation-unambiguous pairs than for translation-ambiguous pairs. The translation norms are made available in a database together with lexical information about the words, which will be a useful resource for researchers investigating Chinese-English bilingual language processing.

**Electronic supplementary material:**

The online version of this article (doi:10.3758/s13428-016-0761-x) contains supplementary material, which is available to authorized users.

## Introduction

Translation equivalents have been used extensively to investigate bilingual language processing. A classic example of the impact of translation equivalents on bilingual language processing is the translation priming effect observed in masked priming experiments. Even though the masked prime is not visible to bilinguals, the recognition of a target word is facilitated by its translation-equivalent non-cognate prime (e.g., Duñabeitia, Dimitropoulou, Uribe-Etxebarria, Laka, & Carreiras, 2010; Duñabeitia, Perea, & Carreiras, 2010; Geyer, Holcomb, Midgley, & Grainger, 2011; Grainger & Frenck-Mestre, 1998; Midgley, Holcomb, & Grainger, 2009). This priming effect also occurs when the two languages do not share the same writing system (e.g., Hebrew-English: Gollan, Forster, & Frost, 1997; Japanese-English: Hoshino, Midgley, Holcomb, & Grainger, 2010; Chinese-English: Wang & Forster, 2010). These findings support the idea of non-selective lexical access (e.g., Dijkstra & van Heuven, 2002; van Heuven & Dijkstra, 1998), which assumes that bilinguals activate both languages when processing words presented in one of their two languages. Additional evidence for non-selective lexical access comes from recent studies using a hidden translation repetition priming paradigm. Thierry and Wu (2007) used this paradigm for the first time to investigate whether translation equivalents are accessed in a purely monolingual context. In their event-related potentials (ERP) study, Chinese-English bilinguals were presented with English word pairs (e.g., *train*-*ham*) and they judged whether the presented word pairs were related in meaning or not. Unknown to the participants, the Mandarin-Chinese (henceforth Chinese) translation of the critical English pairs had a repeated Chinese character with identical pronunciation (e.g., the Chinese translation of *train* and *ham* is **火**车 and **火**腿 respectively, with a repeated character 火). Compared to the non-repeated control condition (e.g., *apple*-*table*, whose Chinese translations are 苹果 and 桌子 respectively), a reduced N400 was found in the repeated character condition. Because no such difference was observed in the ERP data from a group of native English speakers, the reduced N400 was taken as the evidence for the unconscious activation of Chinese translation equivalents. Furthermore, in a masked priming experiment, Zhang, van Heuven, and Conklin (2011) also observed a hidden translation repetition priming effect, suggesting that the unconscious translation is fast and automatic.

A potential issue with the translation priming paradigm is that translation equivalents do not always have one-to-one mappings. When a word in one language has more than one translation equivalent in another language, it is considered to be translation ambiguous. To investigate the prevalence of translation ambiguity, Tokowicz, Kroll, de Groot, and van Hell (2002) asked Dutch-English bilinguals to write down the first translation that came to their mind (first translation method) of words taken from a number of published translation studies (e.g., de Groot, 1992; de Groot, Dannenburg, & van hell, 1994). Surprisingly, more than 25 % of the 562 words in their study that received more than one correct translation were classified in the published translation studies as having an unambiguous translation. Thus, translation ambiguity is in fact more prevalent and studies often incorrectly classify words as translation unambiguous. The prevalence of translation ambiguity is not limited to Dutch and English. In a large-scale Spanish-English translation norming study, more than 50 % of the words were translation-ambiguous (Prior, MacWhinney, & Kroll, 2007). Given that translation ambiguity is common, an important question is whether translation ambiguity affects bilingual lexical access.

Recent studies have revealed that translation ambiguity does indeed influence bilingual language processing (for a review, see Tokowicz, 2014). For example, in a translation production task with English-Spanish bilinguals, Tokowicz and Kroll (2007) found that bilinguals were slower and less accurate when presented with translation-ambiguous words. This disadvantage for translation-ambiguous words has also been reported in word recognition tasks (Boada, Sanchez-Casas, Gavilán, García-Albea, & Tokowicz, 2013; Laxén & Lavaur, 2010). For example, when French-English bilinguals had to judge whether two words were translation equivalents, they responded faster to translation-unambiguous pairs than to translation-ambiguous pairs, and faster to words with their dominant translations than to words with subordinate translations (Laxén & Lavaur, 2010). More surprisingly, as reviewed by Tokowicz (2014), Degani, Prior, Eddington, Arêas da Luz Fontes, and Tokowicz (2013) found that bilinguals showed a disadvantage for translation-ambiguous words even in a monolingual context. In their English lexical decision task, Spanish-English bilinguals’ responses were less accurate for English words with several Spanish translations than for English words with only one Spanish translation. Taken together, these studies not only suggest that translation ambiguity affects language processing but also provide further support for non-selective bilingual lexical access (e.g., Dijkstra, 2005; Dijkstra & van Heuven, 2002; van Heuven & Dijkstra, 1998).

Because of the extensive use of translation equivalents in bilingual studies and the importance of controlling for translation ambiguity, the primary goal of the present study was to create a large database of English-Chinese translation norms and to make this available for other researchers. This database would directly benefit future research using Chinese-English translation equivalents to investigate bilingual language processing. Further research into Chinese-English bilingual language processing is particularly important for a number of reasons. Firstly, although a translation priming effect in masked priming experiments using a lexical decision task has been consistently reported with L1 (Chinese) primes and L2 (English) targets, only one experiment in Jiang (1999) reported a L2-L1 translation priming effect. However, Jiang (1999) failed to replicate this in later experiments. This translation priming asymmetry is a well-known phenomenon in the bilingual literature (for a review, see Dimitropoulou, Duñabeitia, & Carreiras, 2011), but more studies are needed to investigate this asymmetry in Chinese-English bilinguals. Secondly, due to the raising concerns over the replication crisis in psychology (Lindsay, 2015), it is crucial to replicate, for example, the hidden translation repetition priming effect in Chinese-English bilinguals (e.g., Thierry & Wu, 2007; Zhang et al., 2011) with different sets of stimuli. Finally, although several studies provided convincing evidence for the influence of translation ambiguity on bilingual processing (e.g., Boada et al., 2013; Laxén & Lavaur, 2010; Tokowicz & Kroll, 2007), it is unclear whether this finding can be generalized to Chinese-English bilinguals. All the research lines mentioned above cannot be easily advanced without a large database of English-Chinese translation norms.

As far as we are aware, there is only one database reported in the literature that includes English-Chinese translation norms (Tseng, Chang, & Tokowicz, 2014). This translation norming study not only conducted the first in-depth investigation of bilingual participants’ translation errors, but also provided a list of alternative Chinese translations for 562 English words together with semantic similarity ratings for all the translation pairs. However, the norming study does not provide all information about the translations and it has some limitations. Most importantly, no information about how many participants provided each alternative translation is available for words with multiple translations (N = 378). Although there are 165 translation-unambiguous pairs in their database, it is unclear whether the unique translations were generated by the majority of the participants or just by one individual participant. In addition, the language background of the bilinguals was heterogeneous because not all of the bilinguals came from Mainland China where only simplified Chinese is used.

Without a large comprehensive database of English-Chinese translation norms available in the literature, previous studies using, for example, the hidden translation-priming paradigm (Thierry & Wu, 2007; Wu & Thierry, 2010, 2012a, 2012b) repeatedly conducted translation-norming experiments with relatively large groups of bilinguals (N ≥ 10) in which all the intended translations were first checked. Other researchers either recruited a small group of bilinguals (N ≤ 4) to perform a translation norming task or they did not conduct a translation norming study at all (e.g., Guo, Misra, Tam, & Kroll, 2012; Jiang, 1999; Jiang & Forster, 2001; Wang & Forster, 2010, 2015). Translation norming experiments were until now often unavoidable because there was no large scale norming database with English-Chinese translations in the literature.

To fill the gap in the literature and help researchers who are investigating Chinese-English bilingual processing, we obtained translation norms from 28 Chinese-English bilinguals using the “first-translation” method employed in previous translation norming studies (Prior et al., 2007; Tokowicz et al., 2002). However, unlike these previous studies, all bilingual participants in the present study were asked to translate the full list of 1,429 English words, resulting in a large translation database. For each English word, all the correct translations and the number of participants providing the correct translations were included in the database. The most frequent translation was defined as the dominant translation for that English word. Furthermore, we included comprehensive lexical information of the translation equivalents (e.g., word frequency, part of speech and concreteness, see below for details). In addition to collecting translation norms, the present study also investigated the relationship between translation ambiguity and word frequency, concreteness and language proficiency.

Given that the present study focuses on Mandarin Chinese (henceforth Chinese) and English, which differ in many linguistic aspects (for more detailed introduction to Chinese, see Huang, Li, & Simpson, 2014), it is necessary to briefly discuss the linguistic differences between Chinese and English. In terms of the writing system, there is no orthographic overlap between Chinese and English. The alphabetic language English uses letters for writing, whereas Chinese is a meaning-based logography using characters as the basic writing unit. A Chinese character is generally regarded as a morpheme, and consist of spatially marked combinations of strokes. Phonologically, each Chinese character corresponds to a full syllable, which consists of segments (consonants and vowels) and a lexical tone. Mandarin Chinese, unlike English, is a tone language that uses four tones for meaning discrimination. For example, the segment *wu* (written in Pinyin, which is a Romanisation of the Chinese pronunciation) with a high level tone (tone 1, denoted as *wu1*), a rising tone (tone 2, denoted as *wu2*), a falling-rising tone (tone 3, denoted as *wu3*), and a falling tone (tone 4, denoted as *wu4*) can mean “house” (屋 ), “nothing” (无), “five” (五), and “fog” (雾), respectively. As illustrated in these examples of Chinese characters, Chinese is considered to have a deep orthography (Frost, Katz, & Bentin, 1987) because the spelling-sound correspondences are opaque.

### Word frequency and concreteness

In the present study, we first focused on the relationship between word frequency, concreteness, and translation ambiguity. Contradictory findings have been reported in the literature in terms of the relationship between word frequency and translation ambiguity. In regression analyses, both Prior et al. (2007) and Tseng et al. (2014) found that the word frequency of the source language was a reliable predictor of the number of translations. However, whereas a negative correlation between word frequency and number of translations was found for English-Spanish translation pairs (Prior et al., 2007), a positive correlation was observed for English-Chinese translation pairs (Tseng et al., 2014). Thus, in the present study we also investigated the relationship between word frequency and translation ambiguity. Unlike word frequency, the relationship between concreteness and translation ambiguity is more consistent. Using the identical set of English words, the English-Dutch (Tokowicz et al., 2002) and English-Chinese (Tseng et al., 2014) translation norming studies both found a negative correlation between the number of translations and the concreteness ratings obtained from the MRC Psycholinguistic Database (Coltheart, 1981), which indicates that more concrete words have fewer translations. This finding was replicated in a Spanish-English translation norming study which showed that imageability, a lexical characteristic highly correlated with concreteness, was negatively correlated with the number of translations (Prior et al., 2007). However, the MRC Psycholinguistic Database did not provide the concreteness ratings for 18.3 % (103 out of 562) English words in Tokowicz et al. (2002) and Tseng et al. (2014). Therefore, it is important to explore further whether this relationship remains the same when a much larger set of words is used. To estimate word concreteness, we used the concreteness ratings from Brysbaert, Warriner, & Kuperman, (2014). This recent large-scale concreteness database provides concreteness ratings for almost all of the English words used in the present study (99.9 %).

### Second language proficiency

The second focus of our investigation is the role of second language (L2) proficiency on the bilinguals’ translations choices. There are inconsistent findings in the literature about whether L2 proficiency impacts bilinguals’ translation choices. The first study that examined the relationship between the bilinguals’ language proficiency and their translation choices was Prior et al. (2007). In this study the bilinguals’ L2 lexical decision accuracy and their self-assessed rating proficiency were used as estimates of the bilinguals’ L2 proficiency. They found a significant positive correlation between L2 proficiency and bilinguals’ forward translation (L1 to L2) scores, but not their backward translation (L2 to L1) scores. In contrast, Lemhöfer and Broersma (2012) found that bilinguals’ L2 proficiency as measured by an English vocabulary test (LexTALE) was positively correlated with their forward and backward translation accuracy for stimuli taken from Tokowicz et al. (2002). One possible explanation for the inconsistent findings might be the use of different measures of language proficiency. Some studies have suggested that the objective LexTALE vocabulary test is more reliable than self-assessment (Brysbaert, 2013; Khare, Verma, Kar, Srinivasan, & Brysbaert, 2013; Lemhöfer & Broersma, 2012). Although LexTALE has been used as an estimate of L2 proficiency in several studies (e.g., Bultena, Dijkstra, & van Hell, 2014; Diependaele, Lemhöfer, & Brysbaert, 2013; Khare et al., 2013), none of translation norming studies have so far used LexTALE to measure the participants’ language proficiency. Therefore, in the present study the English language proficiency of the bilinguals will be measured using LexTALE (Lemhöfer & Broersma, 2012) as well as self-rated scores of English language skills.

### Dominant and subdominant translations

Because word frequency plays such an important role in monolingual and bilingual language processing (e.g., Antón-Méndez & Gollan, 2010; Dijkstra, Van Jaarsveld, & Brinke, 1998; Kuperman & Van Dyke, 2013; Peeters, Dijkstra, & Grainger, 2013), we expected that word frequency would also impact the bilinguals’ translation choices. To examine this, we first determined the dominant and subdominant translations by searching for the first and second most frequent translations in the present database. If word frequency of the target language does influence the bilinguals’ translation choice, we expected a significant frequency difference between the dominant and subdominant translations.

### Relationship between source and target language

Given that semantic overlap is a key feature of translation equivalents, Tokowicz et al. (2002) and Tseng et al. (2014) used semantic similarity ratings of translation pairs to investigate the relationship between source and target language. With semantic similarity ratings collected from a different group of participants in their translation norming studies, both studies observed that words with more translations were considered less similar to their translation equivalents, indicated by a reliable negative correlation between number of translations and semantic similarity ratings. This observation captures an important fact about translation ambiguity and the bilingual mental lexicon, because the existence of multiple translations may influence bilinguals’ judgments on how similar the translation pairs are in meaning. In the present study, we further explored the relationship of source and target language by investigating the correlation between the word frequencies of the English words and the Chinese translations (dominant translation) and for pairs that are unambiguous and ambiguous. Kondrak (2013) reported a positive correlation between the word frequency of English words and their French translations (r = 0.573), whereas the correlation of word frequency for non-translation pairs was close to zero. Kondrak pointed out that this positive correction for translation pairs was due to the considerable overlap in semantic concepts, which tend to occur with similar frequencies across languages. According to this logic, we expected to find a higher correlation with word frequency for translation-unambiguous pairs than translation-ambiguous pairs. Finally, we examined the correlation of word frequencies among Chinese-English cognate pairs. Chinese-English cognates are transliterated loan words, which were created to keep pronunciations as similar as possible to the original English. Thus, cognates are defined in the present study as loan words with high similarity in phonology between English and Chinese, such as coffee - 咖啡 [kʰa fei].^1^ Because these cross-script cognates can be regarded as semantically unambiguous (Dong & Lin, 2013; Qi, 2011), the correlation of the word frequencies of cognates pairs is likely to be high.

## Method

### Participants

Thirty (Male: 2) Chinese-English bilinguals participated in the study. All participants received an inconvenience allowance. One participant dropped out and another one was excluded from analyses because she did not receive formal Chinese education. The remaining participants included undergraduate (N = 22) and postgraduate (N = 6) university students studying in Nottingham. They met the minimum English language entry requirements to study at the University of Nottingham (IELTS 6.0 for undergraduates and 6.5 for postgraduates). The bilingual participants were all native Mandarin Chinese speakers who learnt Chinese from birth and learnt English by classroom instructions from primary or secondary schools onwards. All participants grew up in Mainland China and were not immersed in an English-speaking environment before they came to the UK for their undergraduate or postgraduate studies. The language background of the bilinguals was comparable to bilinguals who participated in other studies in the literature (Guo et al., 2012; Wang & Forster, 2010, 2015; Wu, Cristino, Leek, & Thierry, 2013; Wu & Thierry, 2010, 2011, 2012a, 2012b; Zhang et al., 2011; Zhou, Chen, Yang, & Dunlap, 2010). For example, participants’ self-rated proficiency scores were similar to those of Zhang et al. (2011), i.e., 4.70 versus 4.69 (7-point scale, 1 = very poor, 7 = native-like ). Table 1 summarizes the participants’ language background data.

**Table 1:** Summary of language background data

	Mean (SD)
Age (years)	22.5 (2.62)
Age exposed to formal English education	9.5 (1.90)
Time studies English (years)	13.0 (2.40)
English immersion experience (months)	11.0 (12.30)
LexTALE test score	57.4 (8.28)
Subjective English ability assessment	
Speaking	4.5 (0.74)
Listening	4.8 (0.70)
Reading	4.9 (0.76)
Writing	4.6 (0.92)

*Note.* LexTALE (Lemhöfer & Broersma, 2012); Speaking, listening, reading and writing ability were rated on a 7-point scale (1 = very poor, 7 = native-like)

### Stimuli

**Fig. 1:**
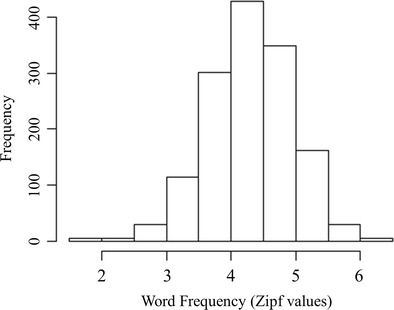
Histogram of English word frequencies (*N* = 1,429)

Because only a few studies in the literature that used English-Chinese translation equivalents provided their experiment stimuli in an appendix (e.g., Dong & Lin, 2013; Guo et al., 2012; Wang, 2013; Wang & Forster, 2015), it is impossible to generate a large set of English words by simply using the experiment stimuli used in previous studies. Therefore, a large set of English words for the current study was selected by translating two-character Chinese nouns from the Contemporary Chinese Dictionary (Institute of Linguistic from Chinese Academy of Social Science, 2005).^2^ Only words that could be translated into a single English word were selected. These Chinese words were considered the expected translations of the English words for the scoring procedures. In total, 1,429 English words were selected with a mean Zipf value of 4.30 (SD = 0.65) in SUBTLEX-UK (van Heuven, Mandera, Keuleers, & Brysbaert, 2014). As shown in Fig. 1, these 1,429 words covered a wide range of frequencies. The mean concreteness rating of the English words (Brysbaert et al., 2014) was 3.69 on a 5-point scale (1 = abstract and 5 = concrete, N = 1,427, SD = 1.06).^3^ The average word length was 6.51 letters (SD = 2.18). Based on the dominant part-of-speech information in SUBTLEX-UK (van Heuven et al., 2014), most of the words were nouns (N = 1,285).

### Procedure

English words were presented one at a time on a computer screen using E-Prime (Schneider, Eschman, & Zuccolotto, 2002). Participants were asked to type in the first translation that came to their mind and they were instructed to skip the word by pressing the ENTER key if they could not translate the word. All participants used simplified Chinese characters when typing Mandarin Chinese words. The 1,429 English words were divided into 14 blocks (about 100 words per block) and the words in each block were matched in frequency. Participants completed the translation task across four separate days within a week. In the first three days, participants completed four test blocks per day. On the final day, after the last two blocks participants completed an English vocabulary test (LexTALE, Lemhöfer & Broersma, 2012) and a language background questionnaire. The 14 blocks were counterbalanced across participants using a Latin Square design. Within each block, the words were presented in a random order. Participants were prompted to take a rest after completing each block. The experiment was approved by the Ethics Committee in the School of Psychology at the University of Nottingham. Participants all signed a consent form before the experiment and were tested individually in a sound-attenuated experimental room.

### Measures of word frequency

As an estimate of word frequency, the present study used the subtitle-based word frequencies from the SUBTLEX-UK (van Heuven et al., 2014) and SUBTLEX-CH (Cai & Brysbaert, 2010). Furthermore, we used the Zipf scale for word frequency. The Zipf scale is a logarithmic scale with values ranging from 1 (very low frequency) to 7 (very high frequency), which are comparable across corpora with different sizes (van Heuven et al., 2014). In addition, the Zipf values for words not available in the databases were calculated using the formula provided by van Heuven et al. (2014), which was 1.47 for SUBTLEX-CH.

### Language proficiency test (LexTALE)

The Lexical Test for Advanced Learners of English (LexTALE) was developed by Lemhöfer and Broersma (2012). In this proficiency test, participants have to decide whether letter strings are real English words or not without any time limit. Although LexTALE only tests English vocabulary knowledge, Lemhöfer and Broersma (2012) demonstrated that the scores are a valid measure of general English proficiency. LexTALE scores range from 0 to 100, and the test is in particular good at differentiating between proficient bilinguals: advanced users (80–100), upper intermediate users (60–80), and lower intermediate users (below 59). LexTALE has become a useful test to quickly measure the participants’ language proficiency (e.g., Bultena et al., 2014; Diependaele et al., 2013; Khare et al., 2013) because it takes less than 5 min to complete and it is freely available (www.lextale.com).

## Results and discussion

### Translation norms

**Fig. 2:**
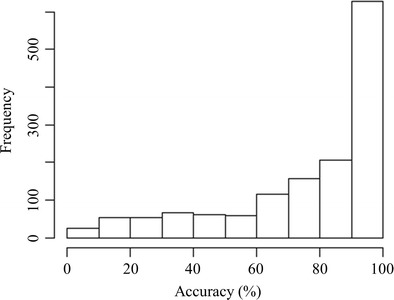
Histogram of the translation accuracy

The Chinese translations provided by the participants were automatically compared to the expected translation in E-Prime. All other translations were manually checked using the Oxford Advanced Learner’s English-Chinese Dictionary (Hornby, 2010). In total, 28 participants provided 35,262 translations for the 1,429 English words. There were 12.0 % (4,750) omitted responses. Out of the 35,262 translations, 86.7 % (30,576) were correct. A large percentage (83.0 %) of the English words (1,187 words) had an accuracy of 50.0 % or more. A histogram of the translation accuracy presented in Fig. 2 revealed that the Chinese-English bilinguals knew most of the English words. The translation accuracy of the English words was positively correlated with word frequency, r_s_ = .601; *p* < .01. This shows that the more frequent the word is, the more likely the meaning was known by the bilinguals.

**Fig. 3:**
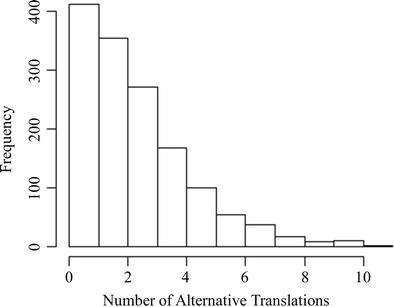
Histogram of the number of translations

Of the 1,429 English words, 0.3 % (five words) received no correct translations, 28.6 % (408 words) received one unique correct translation, and 71.2 % (1,017 words) received more than one correct translation and these were therefore considered translation ambiguous. The histogram of the number of translations is presented in Fig. 3.

For each English word, the most frequent correct translation was defined as the dominant translation for that English word.^4^ The word frequency of the dominant translations also covered a wide range of frequencies (mean = 4.25, SD = 0.74, range 1.47–6.76). Because there were 318 English words in the present study that were also used in Tseng et al. (2014), we further validated the dominant translations by checking whether they are one of the translations provided by participants in Tseng et al. (2014). In total, 96 % of the 318 dominant translations provided for the English words in this study were one of the translations provided for the same English words in Tseng et al. (2014).

The proportion of translation ambiguity in the data (71.2 %) is very similar to the proportion reported in Tseng et al.’s (2014) English-Chinese translation study (67.3 %). Interestingly, compared to same-script language translation norms, the percentage of translation ambiguity is higher for English-Chinese translation pairs than for Dutch-English pairs (Dutch to English: 25.3 %; English to Dutch: 30.4 %, taken from Tokowicz et al., 2002) and for Spanish-English pairs (Spanish to English: 48.1 %; English to Spanish: 57.9 %, taken from Prior et al., 2007). One possible explanation lies in the inherent differences in the writing systems. As mentioned in Tseng et al. (2014), most of the Chinese-English translation pairs received a “1” on a 7-point scale in a form similarity rating task. In contrast, same-script language pairs, such as Dutch-English and Spanish-English have larger orthographic and phonological overlap, which impacts bilinguals’ translation choices because translations similar in form are translated more often than those different in form (Prior et al., 2007). Furthermore, the direction of the translation task may also have contributed to the high percentage of translation ambiguity. In the present study, unbalanced Chinese-English bilinguals performed a backward translation task. Tokowicz et al. (2002) reported that backward translation (L2 to L1) resulted in more alternative translations than forward translation because of the larger vocabulary size in L1. In addition, Chinese is a language full of synonyms or near-synonyms. For example, the English word “surprise”, received three different correct translations in the present study and in Tseng et al. (2014), i.e., “惊喜” (dominant translation), “惊讶”, and “惊奇”. The non-dominant translations could also be translated into synonyms or near-synonyms of its dominant translation, i.e., “诧异”, “惊异”. All these correct translations have very subtle differences in meaning and in the way that they are used in the language so that even native Chinese speakers are sometimes unable to distinguish between them. Given that dialects of Chinese may also vary in vocabulary, the role of synonyms or near-synonyms in Chinese is a complex phenomenon.

### English (L2) word frequency, concreteness, and translation ambiguity

Next, we investigated the relationship between the English word frequency, concreteness, and translation ambiguity. The Spearman’s rank correlation between the number of alternative translations and English word frequency was significant, r_s_ = .161, *p* < .01. This small positive correlation indicated that more frequent words tend to have more translations. This finding is consistent with Tseng et al. (2014), who also reported a small positive correlation between the number of alternative translations and English word frequency (r = .07). However, Prior et al. (2007) reported a negative correlation between word frequency and number of translations when Spanish-English bilinguals translated from Spanish or English. It is not clear why the relationship between word frequency and number of translations is different in the Prior et al. (2007) study. Given that Tseng et al. (2014) and the present study used different sets of English words and reported similar findings, one possible explanation lies in the inherent difference between Chinese and Spanish. As discussed above, Chinese has an overall higher level of translation ambiguity, and is full of synonyms or near-synonyms. Potentially, higher-frequency English words have more Chinese translation equivalents than Spanish translation equivalents.

A Spearman’s rank correlation was also calculated between the number of alternative translations and the concreteness ratings of the English words (N = 1,427) obtained from Brysbaert et al. (2014). Consistent with Tokowicz et al. (2002) and Tseng et al. (2014), a small but significant negative correlation was found, r_s_ = −.190 , *p* < .01. This correlation indicated that more concrete words tend to have fewer alternative translations.

**Fig. 4:**
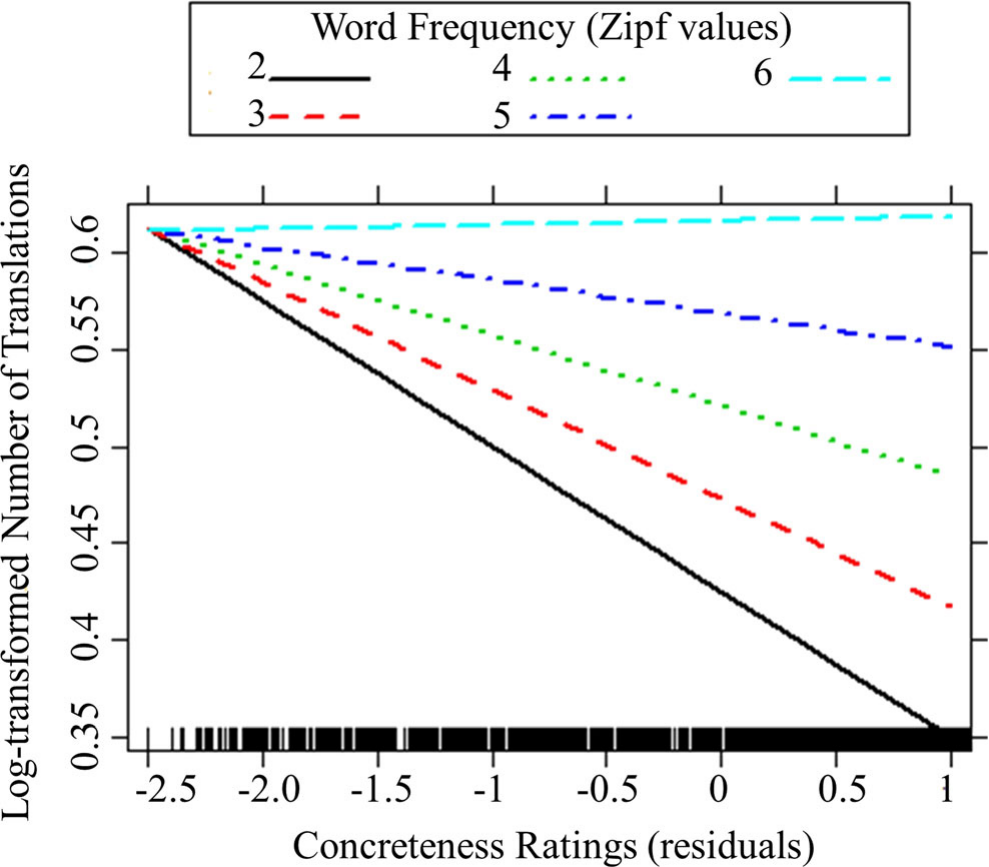
The interaction between word frequency and concreteness ratings

Although correlations analyses suggest a relationship between word frequency, concreteness and translation ambiguity, it is unclear which of these factors predict translation ambiguity. Therefore, a fixed-effects hierarchical regression analysis was conducted using R (version 3.2.4) with the number of translations as the dependent variable and lexical variables of English words as fixed effects. To identify possible predictors, correlation analyses were conducted between the number of translations and other available lexical information: word length and number of part-of-speech categories taken from van Heuven et al. (2014). Both word length and number of part-of-speech categories did not correlate with the number of translations (*p*s > .30). Therefore, the regression analysis considered only word frequency and concreteness ratings as fixed effects. Two English words were excluded from the analysis because they did not have concreteness ratings and five words were excluded because they received no correct translations. The dependent variable (number of translations) was log-transformed (Baayen, 2008). In order to address the issue of the collinearity between word frequency and concreteness ratings, the concreteness ratings were orthogonalised by fitting a linear model in which concreteness ratings were predicted by word frequency (see, for example, Siyanova-Chanturia, Conklin, & van Heuven, 2011, for a similar approach). The residuals of this model were then included in the regression analysis as the concreteness predictor. Word frequency was entered in the first step to predict the number of translations, concreteness ratings were entered in the second step, and their interaction was entered in the final steps. For each step, the model was compared to the previous model using an ANOVA test. We proceeded with the more complex model if the test was significant. In the first step, word frequency (SUBTLEX-UK Zipf values) was a significant predictor. In the second step, word frequency and concreteness ratings were both significant predictors. In the third step, word frequency, concreteness ratings and their interactions were all significant predictors (Table 2). The interaction between word frequency and concreteness ratings was visualized in Fig. 4 using the Effects package (Fox, 2003) in R. As can be seen in Fig. 4, the word frequency effect on number of translations was more pronounced for more concrete words than less concrete words.

**Table 2:** Results of fixed-effects regression analysis

	R^2^, adjusted R^2^	Estimate (SE)	t value	*p*
Step 1	0.02, 002			
Word frequency		0.05 (0.03)	5.97	< .001
Step 2	0.05, 0.05			
Word frequency		0.05 (0.01)	6.06	< .001
Concreteness		-0.31(0.01)	6.59	< .001
Step 3	0.06, 0.06			
Word frequency		-0.16(0.10)	6.23	< .001
Concreteness		-0.11(0.03)	3.46	< .001
Frequency*Concreteness		0.02(0.01)	2.55	< .05

Overall, the regression analysis showed that both word frequency and concreteness ratings significantly predicted the number of Chinese translations for English words. This finding is consistent with previous translation norming studies (Prior et al., 2007; Tseng et al., 2014), which reported that word frequency significantly predicted number of translations. As previously discussed, both Tseng et al. (2014) and the present study found that more frequent words tend to have more alternative translations than less frequent words, whereas Prior et al. (2007) observed the opposite pattern. The regression analyses further revealed that the word frequency effect was more pronounced in more concrete words than less concrete words. In addition, more concrete words tended to have fewer alternative translations than less concrete words, which is in line with the findings of Tokowicz et al. (2002) and Tseng et al. (2014). However, it is important to note that the number of translations is not equal to the number of all the possible translations known to all the participants because the present study and all the previous translation norming studies used the “first-translation” method (the first translation that came to the participants’ mind). Therefore, this methodological limitation requires further research.

### Language proficiency and translation ambiguity

We first obtained the dominant translation scores of each participant by calculating how many times they provided the dominant translations across all the English words. Pearson correlations were then calculated between the participants’ dominant translation scores and the various proficiency measures. Participants’ dominant translation scores were significantly correlated with their LexTALE scores, r = .385, *p* < .05. However, self-rated proficiency scores (listening, speaking, reading and writing) did not correlate with the participants’ dominant translation scores (all *p*s > .10).

To rule out the possibility that more proficient participants were more likely to give any correct translation, the non-dominant translation scores of each participant were calculated by adding up the total number of non-dominant translations provided for all the English words. Participants’ non-dominant translation scores did not significantly correlate with their LexTALE scores, r = .292, *p* = .132 or self-rated proficiency scores (speaking: r_s_ = .362, *p* = .059, other *p*s > .10). We also calculated the participants’ overall translation scores by adding up the total number of all correct translations (dominant and non-dominant translations) provided for all the English words. As expected, participants’ overall translation scores were positively correlated with their LexTALE scores, Pearson’s r = .385, *p* < .05, but not with self-rated proficiency scores (all *p*s > .20). Thus, the bilinguals’ language proficiency did influence their L2 to L1 translation performance when L2 proficiency was measured using LexTALE (Lemhöfer & Broersma, 2012). The positive correlation between the LexTALE scores and dominant translation scores indicated that more proficient bilinguals are more likely to produce dominant translations than less proficient bilinguals. In line with the previous studies (Brysbaert, 2013; Khare et al., 2013; Lemhöfer & Broersma, 2012), self-rated proficiency scores did not correlate with translation performance. A possible explanation is that self-rated proficiency scores are a less sensitive measure of proficiency than objective proficiency measures such as obtained with LexTALE.

As reported above, translation ambiguity (number of translations) at the item level is predicted by word frequency and concretenes. However, it is unclear whether these factors as well as English proficiency influence whether or not participants provided the dominant translation. To investigate this, a mixed-effects logistic regression was conducted using the lme4 package (Bates, Maechler, Bolker, & Walker, 2015) in R. This regression included lexical variables of English words (word frequency, concreteness ratings) and a subject variable (English proficiency) as fixed effects to predict whether participants provided the dominant translation for an English word or not. This analysis is possible because in the current study all bilingual participants were asked to translate all the English words and dominant translations were obtained. Previous studies could not do this analysis because they did not require all participants to translate all the words or they did not provide the dominant translations (Prior et al., 2007; Tokowicz et al., 2002; Tseng et al., 2014).

Participants’ responses were first transformed into a binomial variable: for each English word the response of the participant was coded 1 when their response was the dominant translation and otherwise it was coded as 0. Two English words were excluded from the analysis because they did not have concreteness ratings and five words were excluded from the analysis because they received no correct translations. As before, the collinearity between the word frequency and concreteness ratings was reduced by using the residuals from the linear model. Subjects and items were included in the model as random effects (Baayen, Davidson, & Bates, 2008) and the fixed effects were word frequency, concreteness, and language proficiency (LexTALE scores). A forward model selection procedure was used. For each step, the model was compared to the simpler model using a Chi-squared test. All models that included fixed effects differed significantly from the null mode with only the random effects (*p* < .001). The final model is presented in Table 3. English word frequency, English concreteness ratings and language proficiency were all significant predictors of participants’ translation performance. Any other interactions or the including predictors in the random slopes (Barr, Levy, Scheepers, & Tily, 2013) did not improve the model and did not account for more variance. This final model revealed that participants were more likely to provide the dominant translations for more frequent words and for more concrete words. Furthermore, more proficient bilingual participants tended to provide the dominant translations. Thus, English word frequency, concreteness and language proficiency all predicted participants’ translation performance.

**Table 3:** Results of mixed-effects logistic regression analysis

Random effects	Variance		SD	
Subject	0.16		0.34	
Item	2.32		1.52	
Fixed effects	Estimate	SE	z value	*p*
Intercept	0.73	0.08	9.51	< .001
Word frequency	1.00	0.07	14.80	< .001
Concreteness	0.18	0.04	4.34	< .05
LexTALE scores	0.02	0.01	2.25	
Marginal R^2^	0.08			
Conditional R^2^	0.47			

*Note*. Marginal R^2^ (the proportion of variance explained by the fixed factors alone) and conditional R^2^ (the proportion of variance explained by both the fixed and random factors) were calculated using the function provided on http://jonlefcheck.net/2013/03/13/r2-for-linear-mixed-effects-models/, which is based on Johnson (2014)

### Dominant and subdominant translations

To investigate the effect of the word frequency on the translation choice, 730 English words were selected with a number-of-translations ranging from 2 to 5. The word frequency (Zipf values) of the dominant translations were compared with the subdominant translations in a Wilcoxon Signed Ranks Test. Results revealed that the word frequency difference between the dominant translations and the subdominant translations (4.40 vs. 3.92) was significant, z = 11.29, *p* < .001.^5^ Thus, when participants were asked to translate English (L2) into Chinese (L1) they chose the more frequent Chinese translation equivalents.

### Correlation between source and target language word frequencies

**Fig. 5:**
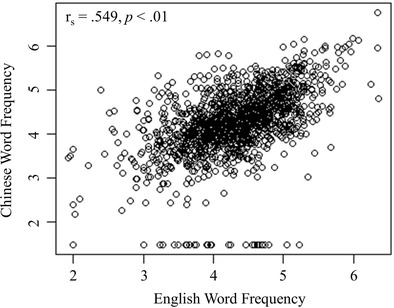
Relationship between English and Chinese word frequency for English words and their dominant Chinese translations

The correlation between the English word frequencies and their dominant Chinese translation frequencies was significant, r_s_ = .549, *p* < .01. This positive correlation (see Fig. 5) indicated that more frequent English words tend to have Chinese translations that are more frequent, even though their writing systems are very different.

**Fig. 6:**
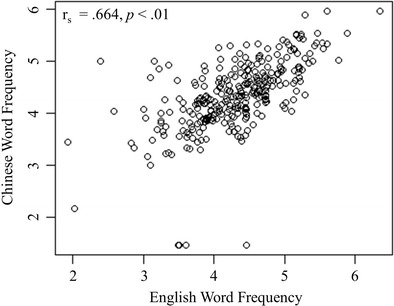
Relationship between English and Chinese word frequency for translation-unambiguous pairs (*N* = 307)

To investigate whether this positive correlation depends on the ambiguity of the translations, the pairs were divided into translation unambiguous and translation ambiguous. In order to identify translation-unambiguous pairs the following criteria were used: (1) one unique correct translation was obtained for the English word, (2) at least 50 % of participants provided that unique translation (the remaining participants either gave no response or an incorrect response), (3) the dominant part-of-speech for the English word was a noun or a name (e.g., bill, prince). This resulted in 307 translation-unambiguous pairs. In total, 560 translation-ambiguous pairs were selected using the following criteria: (1) more than one correct translation was obtained for the English word, (2) at least 50 % of participants provided the dominant translation, (3) the dominant part-of-speech for the English word was a noun or a name. Spearman correlations revealed higher correlations between word frequencies for translation-unambiguous pairs, r_s_ = .664, *p* < .01 (Fig. 6), than for translation-ambiguous pairs, r_s_ = .518, *p* < .01. Importantly, the correlation between the word frequencies of translation-unambiguous pairs were significantly higher than of translation-ambiguous pairs, z = 3.174, *p* < .001 (Diedenhofen & Musch, 2015). This suggests that translation ambiguity impacts the relationship between source and target language. However, some of the translation-unambiguous pairs in the present study were not classified as unambiguous in Tseng et al. (2014). For example, the word “*tail*” is a translation-unambiguous item in our database but ambiguous in Tseng et al. (2014). Furthermore, whereas the word “carrot” was translated into two correct translations in our study it was only translated into one correct translation in Tseng et al. (2014). Therefore, we further investigated the relationship between the translation pairs’ frequencies using cognate pairs, which are considered to be semantically unambiguous.

**Fig. 7:**
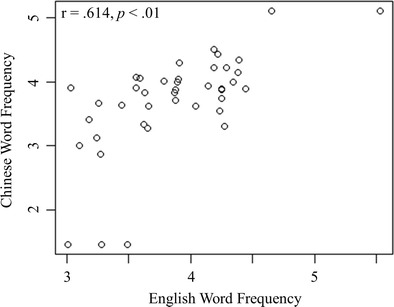
Relationship between English and Chinese word frequency for cross-script cognates (*N* = 41)

Forty-one English-Chinese translation pairs were identified as cross-language cognates using the Contemporary Chinese Dictionary (Institute of Linguistic from Chinese Academy of Social Science, 2005). In this dictionary, transliterated loan words from English are specified together with their original English words. The correlation between the English frequencies and their Chinese cognate translation frequencies was significant, r_s_ = .614, *p* < .01 (Fig. 7). As expected, this correlation was higher than the overall correlation between all the English words and the dominant translations (Fig. 5).

## Summary and conclusions

The present translation norming study revealed that 71 % of the English words were translated into more than one correct translation, and that more frequent words and less concrete words have more alternative translations as revealed by a small but significant correlation. A regression analysis further revealed that English word frequency and concreteness significantly predicted the number of Chinese translations of English words. Furthermore, an interaction between these predictors revealed that the number of translations was more affected by word frequency for more concrete words than for less concrete words. In addition, word frequency, concreteness and bilingual language proficiency were all significant predictors of whether participants provided a dominant translation for an English word or not. An important finding of the present study is that there are significant differences between the word frequency of the dominant translations and the subdominant translations, suggesting that when participants translated English (L2) into Chinese (L1) they selected the more frequent Chinese translation equivalent. Another important finding is the positive correlation between the word frequencies of the English words and their dominant Chinese translations. This positive correlation is higher for translation-unambiguous pairs than for translation-ambiguous pairs, and also high for cross-script cognate pairs.

The present database with backward translation (L2 to L1) norms is useful for future investigations of Chinese-English bilingual language processing, in particular L1 activation during L2 processing. Furthermore, the norms are also be useful for modelling bilingual word recognition and word translation (e.g., Dijkstra & Rekké, 2010) and for cross-language comparisons (e.g., Schepens, Dijkstra, Grootjen, & van Heuven, 2013). A limitation of the current study is that translation norms provided by this database were collected from unbalanced Chinese-English bilinguals conducting backward translation (L2 to L1) only. Therefore, researchers should be cautious when using this database for studies that investigate L2 (English) activation during L1 (Chinese) processing and when the research involves more balanced bilinguals. However, for studies with balanced bilinguals, the backward translation norms provide a baseline. To conclude, the Chinese translation norms for 1,429 English words obtained in this study can be used by researchers investigating Chinese-English bilingual processing. The information provided in the translation database allows bilingual researchers to do cutting-edge investigations without the need for conducting their own norming studies.

## Database

The database with English-Chinese translation norms are available as supplementary information and they can also be downloaded from http://www.psychology.nottingham.ac.uk/ECTN. The database is available in two files. The first file ( ECTN_all_translations.xlsx) contains 23 columns:

1. Word Number (nr)
2. The English word (word)
3. The number of correct alternative translations provided by participants (num_corr_resp)
4. The correct translations provided by participants and number of participants who provided each of the correct translations (e.g., trans1_count is the number for participant provided trans1)

The second file (ECTN_words_results.xlsx) contains 21 columns with the following information:

1. Word Number (nr)
2. The English word (word)
3. The Length of the English word (word_length)
4. The SUBTLEX-UK Zipf value of the English word (word_UK_Zipf)
5. The concreteness rating of the English word (concreteness_Eng)
6. The part-of-speech for the English word (POS_Eng)
7. The dominant part-of-speech for the English word (doPOS_Eng)
8. The dominant Chinese translation for the English word (dom_trans)
9. The number of characters in the dominant Chinese translation (dom_trans_nchar)
10. The number of strokes in the dominant Chinese translation (dom_trans_stroke)
11. The SUBTLEX-CH Zipf value of the dominant Chinese translation (dom_trans_Zipf)
12. The part of speech for the dominant Chinese translation (dom_trans_POS)
13. The dominant part of speech for the dominant Chinese translation (dom_trans_doPOS)
14. Pinyin with tone of the dominant Chinese translation (dom_trans_pinyin)
15. IPA of the dominant Chinese translation (dom_trans_IPA)
16. The cognate/loan word status (dom_trans_cognate)
17. The percentage of the dominant translation (percent_dom_trans)
18. The percentage of correct translations (percent_correct)
19. The percentage of incorrect translations (percent_incorrect)
20. The percentage of omitted responses (percent_omitted)
21. The number of correct alternative translations provided by participants (num_corr_resp)

*Note*: The SUBTLEX-UK Zipf values, part-of-speech, and dominant part-of-speech of the English words were taken from van Heuven et al. (2014); concreteness ratings of the English words were taken from Brysbaert et al. (2014); SUBTLEX-CH Zipf values, part-of-speech and dominant part of speech of the dominant Chinese translations were taken from Cai and Brysbaert (2010); IPA of the dominant Chinese translation was taken from Zhao and Li (2009).

## Electronic supplementary material

Below is the link to the electronic supplementary material.

ESM 1 (XLSX 253 kb)

ESM 2 (XLSX 108 kb)

## Footnotes

1 Cognates have generally been defined in same-script language pairs as words that “have (almost) the same form and meaning” (Dijkstra, 2007, p. 373). Recently, cognates have been defined as words that “share all or a large part of their phonological and/or orthographic form with their translation in another language” (de Groot, 2011, p. 449). Studies involving different-script languages have used the term “cognate” to refer to phonologically similar translation pairs (e.g., Hebrew-English: Gollan et al., 1997; Japanese-English: Hoshino & Kroll, 2008; Korean-English: Kim & Davis, 2003; Greek-English: Voga & Grainger, 2007). As pointed out by Voga and Grainger (2007), the notion that cognates are phonologically and orthographically similar translations is “not a viable definition for languages with different scripts” (p. 948). In line with these studies, we defined in our manuscript cognates as phonologically similar translations.

2 The Contemporary Chinese Dictionary is known as 现代汉语词典 (Xiandai Hanyu Cidian) and compiled by the Institute of Linguistic from Chinese Academy of Social Science. This is a well-known and authoritative dictionary in Mainland China.

3 British English was used in the study, whereas the concreteness ratings are based on American English. Therefore, we obtained the concreteness ratings for 15 British English words using their American counterparts: millimetre (millimeter), centimetre (centimeter), theatre (theater), fibre (fiber), saviour (savior), neighbour (neighbor), behaviour (behavior), flavour (flavor), colour (color), labour (labor), harbour (harbor), humour (humor), pyjamas (pajamas), cheque (check), defence (defense). In addition, the concreteness ratings for 6 words were obtained from their corresponding singular/plural: criterion (criteria), nobles (noble), jungles (jungle), materials (material), maths (math), fans (fan).

4 Forty-six items have two translations with equal participants. In these cases we used the expected translation as the dominant translation. The file ECTN_all_translations.xlsx contains all the correct translations and the number of participants providing each translation.

5 We also conducted a further analysis using 289 English words with two alternative translations. Results also revealed that the word frequency difference between the dominant translations and the subdominant translations (4.53 vs. 3.89) was significant, z = 9.89, *p* < .001.
